# Clinico-microbiological profile of *Burkholderia cepacia* keratitis: a case series

**DOI:** 10.1186/s12941-020-00407-6

**Published:** 2021-01-07

**Authors:** Ming-Chih Ho, Eugene Yu-Chuan Kang, Lung-Kun Yeh, David H. K. Ma, Hsin-Chiung Lin, Hsin-Yuan Tan, Hung-Chi Chen, Ching-Hsi Hsiao

**Affiliations:** 1 Department of Ophthalmology, Chang Gung Memorial Hospital, No. 199, Tung-Hwa North Road, Linkou, Taipei, Taiwan; 2 College of Medicine, Chang Gung University, Taoyuan, Taiwan

**Keywords:** *Burkholderia cepacia*, keratitis, corneal ulcer, drug susceptibility

## Abstract

**Background:**

*Burkholderia cepacia*, an opportunistic pathogen mainly affecting patients with cystic fibrosis or immunocompromised, has rarely been documented as a cause of corneal infection. The clinical and microbiological profiles of *B. cepacia* keratitis are reported herein.

**Methods:**

We retrospectively reviewed the medical record of 17 patients with culture-proven *B. cepacia* keratitis, treated between 2000 and 2019 at Chang Gung Memorial Hospital, Taiwan. Our data included predisposing factors, clinical presentations, treatments, and visual outcomes of *B. cepacia* keratitis as well as the drug susceptibility of the causative agent.

**Results:**

The most common predisposing factor for *B. cepacia* keratitis was preexisting ocular disease (seven, 41.2%), particularly herpetic keratitis (five). Polymicrobial infection was detected in seven (41.2%) eyes. All *B. cepacia* isolates were susceptible to ceftazidime. Main medical treatments included levofloxacin or ceftazidime. Surgical treatment was required in five (29.4%) patients. Only four (23.5%) patients exhibited final visual acuity better than 20/200.

**Conclusions:**

*B. cepacia* keratitis primarily affects patients with preexisting ocular disease, particularly herpetic keratitis, and responds well to ceftazidime or fluoroquinolones. However, the visual outcomes are generally poor.

## Background

*Burkholderia cepacia* complex, formerly known as *Pseudomonas cepacia*, is a group of aerobic Gram-negative bacilli comprising more than 20 species [1, 2]. It can exist in various environments, such as soil or water, and can infect both humans and plants. In humans, *B. cepacia* is principally an opportunistic pathogen that causes various diseases, such as lung infections, in patients with cystic fibrosis or chronic granulomatous disease. Ocular manifestations caused by *B. cepacia* include endophthalmitis and keratitis, both of which are vision-threatening [3–9]. Several case series have reported on *B. cepacia* endophthalmitis, which occurs after ocular surgery or ocular trauma. Compared with endophthalmitis, *B. cepacia* keratitis has rarely been reported, with only eight sporadic cases being documented thus far [4, 5, 7, 9, 10]. Here, we report on 17 cases of *B. cepacia* keratitis. By reviewing patient demographics, risk factors, clinical presentations, treatment, and visual outcomes, we identified the characteristics of the disease, thus contributing additional knowledge on *B. cepacia* keratitis.

## Materials and methods

This single-center retrospective study included data of 17 patients diagnosed as having *B. cepacia* keratitis at Chang Gung Memorial Hospital, Taiwan between December 2003 and August 2019. Corneal scrapings, obtained under topical anesthesia, were inoculated on blood and chocolate agar, thioglycolate broth, and Lowenstein–Jenson agar as well as subjected to Gram staining. The various media were routinely incubated for one week or longer, depending on the medium, before the final culture result was obtained. Isolates were identified, by using conventional biochemical tests; matrix-assisted laser desorption/ionization time-of-flight (MALDI-TOF) mass spectrometry was applied starting in 2013. Antimicrobial susceptibility was evaluated using the standard disk diffusion method and interpreted according to the guidelines established by the Clinical and Laboratory Standards Institute (CLSI). For *B. cepacia* isolates, ceftazidime, meropenem, and sulfamethoxazole–trimethoprim were tested. Each patient’s demographic data, risk factors, clinical presentations, treatments, and visual outcomes were reviewed. We provided a case report (patient 13) as a representative of *B. cepacia* keratitis in our study. We defined the location of an ulcer as central if it was located within 2 mm of fixation, periphery if it involved a zone within 2 mm from the limbus, and paracentral if it was in between. The ulcer size was defined as small (< 2 mm), medium (2–6 mm), or large (> 6 mm) on the basis of the longest diameter. Predisposing factors were classified into ocular trauma, contact lens wear, preexisting ocular disease, recent ocular surgery, and systemic disease. Prior steroid use was also recorded. Visual acuity was measured using Snellen charts.

## Results

Table 1 lists the demographic and clinical data of the patients. The mean patient age was 62.4 ± 17.2 (range 24–88) years. A total of 17 eyes were involved, with nine right eyes and eight left eyes in eight male and nine female patients. Mean follow-up duration was 2.76 years (range 7 days to nine years).

**Table 1:** The demographic and baseline characteristics of the patients with *Burkholderia capecia* keratitis

No.	Age/ sex	Year	Risk factors	Prior use of cortico- steroid	Location , size	Hypopyon	Corneal perforation	Treatment	Surgery	Presenting VA	Final VA	Other isolates
1	71/f	2003	Diabetic mellitus	−	C, L	+	−	Ceftazidime, Vancomycin	−	NLP	NLP	
2	52/m	2007	Ocular trauma	+	C, S	+	−	Ceftazidime, Sulfamethoxazole/Trimethoprim	AMT	CF	CF	*Candida parapsil*osis
3	64/f	2010	Diabetic mellitus, Recent ocular surgery (PKP)	+	C, L	−	−	Ceftazidime	AMT, patch graft, evisceration	NLP	NLP	
4	71/m	2010	HZV keratitis	−	C, L	−	+	Amikacin, Vancomycin, Sulfamethoxazole/Trimethoprim	−	NLP	NLP	*Serratia marcesc*ens
5	24/m	2010	HSV keratitis	+	PC, L	−	−	Ceftazidime, acyclovir	−	CF	20/200	
6	57/f	2014	HSV keratitis	−	C, L	−	+	Levofloxacin, acyclovir	Patch graft, PKP	HM	LP	
7	85/m	2016	Unknown	−	C, L	+	−	Amikacin, voriconazole	−	HM	HM	*Fusarium solani*
8	53/m	2016	Ocular trauma	+	PC, M	+	+	Ceftazidime, Moxifloxacin (oral), Vancomycin		20/200	20/70	
9	84/f	2017	Ocular surface problem	−	PC, S	−	−	Levofloxacin	−	CF	CF	
10	50/f	2017	Contact lens wear	−	PC, S	−	−	Levofloxacin	−	20/25	20/50	
11	88/f	2017	Recent ocular surgery (PKP, AMT)	+	C, M	+	−	Levofloxacin	AMT	HM	20/1000	*Pseudomonas aeruginosa*
12	60/m	2017	Unknown	−	C, L	+	−	Levofloxacin	AMT, keratectomy, tarsorrhaphy, patch graft	CF	NA	
13	84/f	2018	HSV keratitis	+	PC, S	+	+	Ceftazidime	−	HM	CF	
14	65/f	2018	Diabetic mellitus, Recurrent ocular ulcer	+	PC, M	+	−	Levofloxacin	−	HM	NLP	*Corynebacterium propinquum, Corynebacterium species*
15	48/m	2018	Ocular trauma	−	PC, S	−	−	Levofloxacin	−	20/50	20/30	*Bacillus megaterium*, *Arthrobacter species*
16	69/f	2019	Unknown	−	C, L	−	−	Levofloxacin	−	NLP	NLP	*Pseudomonas aeruginosa*
17	36/m	2019	HZV keratitis, Contact lens wear	+	C, M	−	−	Levofloxacin	−	CF	20/400	

*AMT * amniotic membrane transplantation, *C * central, *CF * counting fingers, *f * female, *HM * hand motion, *HSV * herpes simplex virus, *HZV * herpes zoster virus, *L * large, *LP * light perception, *m * male, *M * medium, *NA * not available, *NLP * no light perception, *PC * paracentral, *PKP * penetrating keratoplasty, *S * small, *VA * visual acuity

Of the 17 corneal ulcers, 10 (58.8%) were located in the central cornea. In terms of size, eight (47.1%), four (23.5%), and five (29.4%) corneal ulcers were defined as large, medium, and small, respectively. Hypopyon was present in eight (47.1%) patients. Corneal perforation was observed in four (23.5%) patients—in two at presentation and in two during treatment.

Predisposing factors of keratitis were identified in 14 patients, with four patients demonstrating multifactorial causes of keratitis. Preexisting ocular diseases (seven eyes, 41.2%), particularly herpetic keratitis (five eyes), was the most common predisposing factor. Other risk factors, including trauma (three eyes), systemic disease (three eyes), contact lens wear (two eyes), and recent ocular surgery (two eyes), were relatively evenly distributed. Prior corticosteroid use was noted in eight (47.1%) patients.

Of the 17 *B. cepacia* culture-positive scrapings, seven cases (41.2%) were polymicrobial (Table 1). All 17 *B. cepacia* isolates were susceptible to ceftazidime; all except for one (16/17, 94.1%) were susceptible to meropenem and sulfamethoxazole–trimethoprim.

All patients were treated with empiric topical antibiotics initially, and adjustments were made according to clinical response or culture results. Levofloxacin, ceftazidime, and amikacin, the main antibiotics for treating *B. cepacia* keratitis, were prescribed to nine (52.9%), six (35.3%), and two (11.7%) patients, respectively. In patients with polymicrobial keratitis, other antimicrobials were added. A total of 12 patients (70.6%) responded well to antimicrobials, whereas five patients (29.4%) required surgical interventions including amniotic graft transplantation, patch graft, tarsorrhaphy, and evisceration. Multiple surgeries were required in three patients.

Visual acuity (VA) worse than 20/200 was noted in 14 patients (82.4%) at presentation; moreover, four patients (23.5%) had no light perception. After treatment, six eyes exhibited improved vision but only four patients (23.5%) had a final VA better than 20/200.

### **Case report** (Patient 13)

**Fig. 1:**
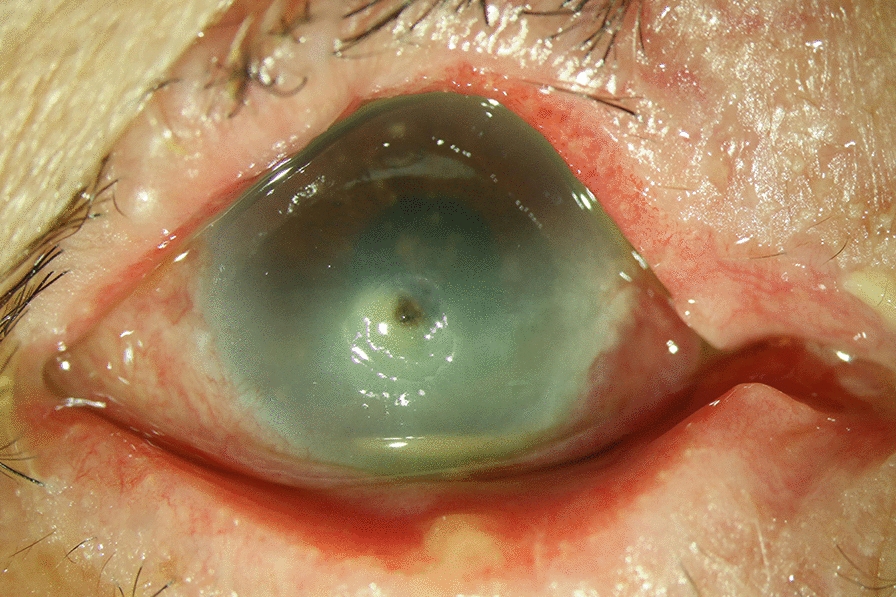
The slit-lamp photograph revealed central corneal epithelial defect with infiltrate, thinning with a descemetocele, and hypopyon

An 84-year-old female patient with herpes simplex virus disciform keratitis was undergoing treatment with topical prednisolone acetate (1%) and oral acyclovir and exhibited sudden onset of blurred vision in her right eye one month after discontinuing the antiviral medication. On examination, VA in the right eye was hand motions. Slit-lamp examination revealed corneal epithelial defect with infiltrate, thinning with a descematocele, and localized edema; strong anterior chamber reaction with hypopyon was also present (Fig. 1). Corneal scrapings were sent for cultures. She was administered on topical vancomycin (25 mg/mL) and ceftazidime (25 mg/mL) hourly and oral famciclovir three times a day. The corneal culture grew *B. cepacia* complex, susceptible to ceftazidime, meropenem, and sufamethoxazole-trimethoprim. She was maintained on topical ceftazidime, and when the infection was controlled, a topical corticosteroid was added. The ulceration resolved within 1 week. At 9-month follow-up, she had a corneal scar with VA of 20/400 in the right eye.

## Discussion

*B. cepacia* is a rare causative agent of keratitis; only eight cases of *B. cepacia* keratitis have been reported in previous studies (Table 2). *B. cepacia* accounted for 0.51% (5/875) of microbial keratitis cases in our previous ten-year (2003–2012) study [11], but we identified 12 more cases in recent years. To our best knowledge, this study is by far the largest case series related to *B. cepacia* keratitis. In conjunction with previously reported cases, we provided a more detailed overview of the clinical characteristics of *B. cepacia* keratitis.

**Table 2:** Clinical data of the patients with *Burkholderia capecia* keratitis

	Age/sex	Risk factor	Prior steroid use	Location, size	Hypopyon	Medical treatment	Surgery	Presenting VA	Final VA	Other isolates\
Matoba et al. [7]	59/m	HSV keratitis	+	C, M	-	Levofloxacin	-	CF	20/200	*Enterococcus* species, *Staphylococcus aureus*
Lin et al. [5]	16/f	Ortho-keratology lens	–	PC, S	–	Ciprofloxacin	–	20/40	20/20	*Pseudomonas putida*, *Pseudomonas aeruginosa*.
Ornek et al. [18]^a^	78/f	Cataract surgery	+	C, M	–	Ciprofloxacin +IVI Ceftazidime	–	NLP	NA	
Chaurasia et al. [4]	NA	Unknown	+	NA, M	–	Ciprofloxacin	Tissue adhesive	LP	NA	
	NA	Unknown	–	NA, S	+	Ciprofloxacin	–	NA	NA	
	NA	Trauma	–	NA, L	+	Ciprofloxacin	TPK	LP	NA	
	NA	TPK	*+*	NA, S	–	Ceftazidime	–	HM	NA	
Reddy et al. [9]	27/m	LASIK	+	C, multiple infiltrates	+	Tobramycin and Gatifloxacin		CF	20/20	

*C * central, *CF * counting fingers, *f * female, *HM * hand motion, *IVI * intravitreal injection, *L * large, *LASIK * laser assisted in situ keratomileusis, *LP * light perception, *m * male, *M * medium, *NA * not available, *NLP * no light perception, *PC * paracentral, *S * small, *TPK * therapeutic penetrating keratoplasty

^a^ A case of keratitis and endophthalmitis

In our study, the most common predisposing factor of *B. cepacia* keratitis was preexisting ocular disease, particularly herpetic keratitis. Matoba et al. also presented a patient with herpetic stroma keratitis, under oral acyclovir and topical prednisolone acetate treatment, who developed polymicrobial keratitis including *B. ambifaria* (belonging to the *B. cepacia* complex), *Enterococcus* spp., and *Staphylococcus aureus* [7]. Infection with herpes virus might cause sub-basal nerve damage of the cornea [12, 13]. The impaired corneal sensory innervation leads to a reduction of protective reflexes and trophic neuromodulators, which affect the wound-healing function of the cornea [14], making its surface an easy target for opportunistic bacteria such as *B. cepacia*. In addition, if the local immune response has been suppressed by topical steroids, a herpetic corneal ulcer can predispose microbial adherence, furthering the infection. Recent ocular surgery with simultaneous topical steroid use was noted in three of the previously reported eight patients with *B. cepacia* keratitis and two patients in our study (Tables 1 and 2), suggesting that local immunosuppression may play a role in such an opportunistic infection.

In our study, approximately 40% of *B. cepacia* culture-positive corneal scrapings were polymicrobial, as were two (25%) of the previously reported eight cases (Table 2). These mixed infections might be due to direct inoculation because of a corneal injury, contamination through the process of corneal scraping, or opportunistic transmission in these immunocompromised patients [15]. Tuft et al. proposed a synergy effect of interactions between organisms in polymicrobial infection [16] and speculated that the primary organism may create a niche, either by providing a sequestered environment or by supplying specific metabolic requirements for a second organism, that predisposes the host to further infection or turns a normally nonpathogenic organism into a pathogen. The mixed infections might modulate the clinical course of the disease, causing unexpected treatment effects.

*B. cepacia* demonstrates multidrug resistance, including resistance to carboxypenicillins, polymyxins, and aminoglycosides. Nevertheless, sulfamethoxazole–trimethoprim, ceftazidime, and meropenem have been revealed to be the most effective agents on the basis of *in vitro* susceptibility data, which agrees with our drug susceptibility test results [17]. We did not test for susceptibility to fluoroquinolones, the most popular empiric antibiotic in the field of ophthalmology. Chaurasia et al. performed an antibiotic susceptibility test for four *B. cepacia* isolated from keratitis and reported 100% susceptibility to ceftazidime and 50% susceptibility to ciprofloxacin/norfloxacin [4]. In the case report by Reddy et al. the isolate from the patient with *B. cepacia* keratitis was resistant to moxifloxacin, gatifloxacin, tobramycin, and ceftazidime and susceptible only to sulfamethoxazole–trimethoprim *in vitro*; nevertheless, *in vivo*, the ulcer resolved completely after tobramycin and gatifloxacin treatment (Table 2) [9]. The other three isolates from previously reported *B. cepacia* keratitis cases were susceptible to ceftazidime and ciprofloxacin [5, 7, 18]. On the basis of the antibiotic susceptibility and clinical results of the patients with *B. cepacia* keratitis (Tables 1 and 2), fluoroquinolones could be initiated as empiric antibiotics. However, if fluoroquinolone use does not improve the clinical course, ceftazidime may be a suitable alternative. Even after aggressive medical treatment, about one-third of the patients in our study and two (25%) of the previously reported eight *B. cepacia* keratitis cases required surgical interventions (Tables 1 and 2).

The visual outcome of *B. cepacia* keratitis was generally poor both in our and previously reported cases (Tables 1 and 2). The unfavorable visual outcomes may be related to old age, poor vision at presentation, comorbidities, and mixed infections. The rather high surgical rates and perforation rates may also contribute to the poor prognosis of the disease.

The retrospective design and small sample size are the limitations of this study. In addition, elucidating the real pathogenic role of *B. cepacia* was difficult because polymicrobial infections were detected in approximately 40% of our patients. Nevertheless, as the largest case series reporting *B. cepacia* keratitis, this study provides more detailed information regarding the clinical and microbiological profiles of this infection.

In conclusion, although relatively uncommon, *B. cepacia* could be a causative agent of infectious keratitis. Our findings revealed that preexisting ocular disease, particularly herpetic keratitis, was the leading predisposing factor of *B. cepacia* keratitis. *B. cepacia* demonstrated clinical response to the treatment of ceftazidime and fluoroquinolone, but some patients required surgical intervention. However, the visual outcome was generally poor.

## Data Availability

The data analyzed during this study are available on request from the corresponding author, Ching-Hsi Hsiao. The data are not publicly available due to it containing information that could compromise the privacy of research participants.

## Abbreviation

VA: Visual acuity
